# The diagnostic and prognostic values of plasma Epstein-Barr virus DNA for residual cervical lymphadenopathy in nasopharyngeal carcinoma patients: a retrospective study

**DOI:** 10.1186/s40880-019-0357-9

**Published:** 2019-03-29

**Authors:** Sai-Lan Liu, Xue-Song Sun, Xiao-Yun Li, Lin-Quan Tang, Qiu-Yan Chen, Huan-Xin Lin, Yu-Jing Liang, Jin-Jie Yan, Chao Lin, Shan-Shan Guo, Li-Ting Liu, Yang Li, Hao-Jun Xie, Qing-Nan Tang, Hu Liang, Ling Guo, Hai-Qiang Mai

**Affiliations:** 1 State Key Laboratory of Oncology in South China, Collaborative Innovation Center for Cancer Medicine, Guangdong Key Laboratory of Nasopharyngeal Carcinoma Diagnosis and Therapy, Sun Yat-sen University Cancer Center, Guangzhou, 510060 Guangdong P. R. China; 2 Department of Nasopharyngeal Carcinoma, Sun Yat-sen University Cancer Center, Guangzhou, 510060 Guangdong P. R. China; 3 Department of Radiation Oncology, Sun Yat-sen University Cancer Center, Guangzhou, 510060 Guangdong P. R. China

**Keywords:** Nasopharyngeal carcinoma, Residual cervical lymphadenopathy, Prognosis, Epstein-Barr virus, Fine needle aspiration cytology, Survival

## Abstract

**Background:**

Currently, the diagnosis and treatment of nasopharyngeal carcinoma (NPC) patients with residual cervical lymphadenopathy following radical radiotherapy with or without chemotherapy are challenging. We investigated the prognosis of NPC patients with residual cervical lymphadenopathy and assessed the diagnostic and prognostic values of Epstein-Barr virus (EBV) DNA in these patients.

**Methods:**

This study included 82 NPC patients who were diagnosed with suspected residual cervical lymphadenopathy following completion of antitumor therapy. Their plasma EBV DNA levels were measured using quantitative polymerase chain reaction (qPCR) before the initiation of treatment and before neck dissection. Fine needle aspiration cytology (FNAC) was performed in 21 patients. All patients had undergone neck dissection and postoperative pathological examination to identify the nature of residual cervical lymphadenopathy. The overall survival (OS), progression-free survival (PFS), distant metastasis-free survival (DMFS), and locoregional relapse-free survival (LRRFS) were calculated using the Kaplan–Meier method and compared using the log-rank test. The Cox proportional hazards model was used to calculate hazard ratios (HRs) with 95% confidence intervals (CIs). Multivariable analysis was used to estimate the effect of potential prognostic factors on survival.

**Results:**

Following a median follow-up of 52.6 months, compared with patients with negative postoperative pathological findings for residual cervical lymphadenopathy, the patients with positive findings had a significantly lower 3-year PFS rate (49.9% vs. 83.3%, *P *= 0.008). Among NPC patients with residual cervical lymphadenopathy, the patients with preoperative plasma EBV DNA > 0 copy/mL had a lower 3-year PFS rate than did those with no detectable EBV DNA (43.7% vs. 61.1%, *P *= 0.031). In addition, combining FNAC with preoperative EBV DNA detection improved the diagnostic sensitivity. Multivariable analysis demonstrated that residual cervical lymphadenopathy with positive postoperative pathological result was an independent prognostic factor for PFS and that detectable preoperative plasma EBV DNA was an independent prognostic factor for OS.

**Conclusions:**

Using FNAC combined with preoperative EBV DNA detection improves the sensitivity in diagnosing NPC with residual cervical lymphadenopathy. Compared with patients with undetectable EBV DNA, patients with detectable preoperative plasma EBV DNA have worse prognosis and may require a more aggressive treatment strategy.

## Background

Nasopharyngeal carcinoma (NPC) differs from malignant tumors arising from other head and neck mucosal sites in epidemiology, pathological types, and treatment [1]. NPC has a distinct ethnic and geographical distribution in Guangdong, South China, where environmental factors, genetic predisposition, and Epstein-Barr virus (EBV) infection play important roles in its pathogenesis. Radiotherapy is the primary treatment of NPC. Several prospective randomized trials [2–5] and meta-analyses [6–8] have demonstrated that concurrent chemoradiotherapy (CCRT) with or without adjuvant chemotherapy (AC) is superior to radiotherapy alone for treating NPC. Currently, intensity-modulated radiotherapy (IMRT) is the preferred irradiation technique for NPC. Although it provides excellent locoregional control [9], a small proportion of patients have residual cervical lymphadenopathy following radical radiotherapy with or without chemotherapy [10]. The treatment of these patients is challenging. According to our previous study [11], approximately 3% of patients had residual cervical lymphadenopathy following IMRT, which is consistent with another report [12]. The National Comprehensive Cancer Network (NCCN) guidelines advocate neck dissection for these patients [13], with well-proven efficacy and safety [14–18]. This group of patients faces a clinical dilemma. Because lymphadenopathy may harbor disease or merely appear as post-treatment necrosis or hyaline fibrosis without viable tumor cells [14–16, 19], it may be difficult to define the nature of the cervical masses due to post-irradiation changes in neck soft tissues [15, 18, 19]. Furthermore, since most studies have focused on the efficacy and safety of neck dissection in this group of patients, there is no commonly accepted method for post-treatment determination of malignancy.

The plasma EBV DNA level has been the most effective predictive biomarker in guiding the treatment and predicting the prognosis of NPC [20]. NPC patients with high levels of EBV DNA before treatment have a high risk of disease recurrence and distant metastasis [21, 22]. However, there is a paucity of data addressing the efficacy of plasma EBV DNA level in determining the diagnosis and prognosis of NPC patients with residual cervical lymphadenopathy.

In this study, we reviewed the clinical charts of NPC patients who were diagnosed with suspected residual cervical lymphadenopathy following radical definitive radiotherapy to assess the diagnostic and prognostic values of plasma EBV DNA level. Furthermore, we analyzed the prognosis of these patients based on preoperative plasma EBV DNA levels.

## Patients and methods

### Design, setting, and participants

For this retrospective study, we collected data from NPC patients who had been diagnosed with suspected residual cervical lymphadenopathy following completion of radical radiotherapy with or without chemotherapy. This study was approved by the Institutional Review Board of the Sun Yat-sen University Cancer Center. Patients were eligible for this study if they fulfilled all of the following criteria: (1) newly diagnosed NPC without metastasis; (2) biopsy-proven World Health Organization type II/III NPC [23]; (3) no history of previous antitumor therapy; (4) completion of radical radiotherapy with or without chemotherapy; (5) lymph nodes that persisted for about 3 months after the completion of antitumor therapy; (6) no local tumor residue or distant metastasis detected before neck dissection; (7) neck dissection at the Department of Head and Neck Surgery; and (8) postoperative pathological examination at Sun Yat-sen University Cancer Center between January 2006 and December 2014. NPC patients with neck recurrence (i.e., reappearance of lymphadenopathy after complete regression of initial lymphadenopathy) [17] or residual cervical lymphadenopathy patients who underwent chemotherapy or salvage re-irradiation alone were excluded. Clinical, pathological, and radiological data of eligible patients were reviewed and reclassified. All patients were restaged according to the 7th edition of American Joint Committee on Cancer/Union for International Cancer Control (AJCC/UICC) staging system.

### Quantification of plasma EBV DNA levels

The plasma EBV DNA levels of patients were measured using quantitative polymerase chain reaction (qPCR) before the initiation of treatment and before neck dissection as described in a previous study [24]. The real-time qPCR system was developed at the *Bam*HI-W region. The system consisted of the amplification primers W-44F (5ʹ-AGTCTCTGCCTCCAGGCA-3ʹ) and W-119R (5ʹ-ACAGAGGGCCTGTCCACCG-3ʹ) and the dual-labeled fluorescent probe W-67T (5ʹ-[FAM]CACTGTCTGTAAAGTCCAGCCTCC[TAMRA]-3ʹ). The β-actin gene was used as a loading control, and the primers 5ʹ-ACAGGCACCAGGGCGTGATGG-3ʹ (forward) and 5ʹ-CTCCATGTCGTCCCAGTTGGT-3ʹ (reverse) and the dual-labeled fluorescent probe sequence 5ʹ-[FAM]CATCCTCACCCTGAAGTACCCCATC[TAMRA]-3ʹ were used.

The cutoff value of plasma EBV DNA level before neck dissection was based on a detectable/undetectable status (0 copy/mL), whereas the cutoff value before the initiation of treatment was as previously established (4000 copies/mL) [21, 25]. No attempt was made to perform repeated analyses using alternative cutoffs.

### Clinical assessment

All patients were evaluated with a complete physical examination, fiberoptic nasopharyngoscopy, magnetic resonance imaging (MRI) or computed tomography (CT) of the head and neck, electrocardiography, positron emission tomography/computed tomography (PET/CT) or chest radiography plus abdominal ultrasonography and bone scan by emission computed tomography, complete blood count with differential counts, biochemical profile, and plasma EBV DNA detection before treatment.

All patients diagnosed with NPC were treated with conventional radiotherapy or IMRT. Whether chemotherapy was administered depended on patient’s age and the stage of disease. All patients with suspected residual cervical lymphadenopathy underwent a preoperative EBV DNA detection and neck dissection. In clinical practice, the gold standard method for determining the nature of residual cervical lymphadenopathy of NPC patients is pathological examination of neck dissection specimens. Therefore, we compared the sensitivity and specificity of fine needle aspiration cytology (FNAC) and preoperative plasma EBV DNA detection with postoperative pathological examination.

### Follow-up

Patients were assessed at the time of treatment completion, and then at least every 3 months over the next 3 years and at least every 6 months thereafter. The evaluation of patients at follow-up included a clinical examination, nasopharyngeal endoscopy, MRI of the nasopharynx and neck area, chest radiography, and abdominal ultrasonography. Patients’ statuses were determined by reviewing their medical records and follow-up findings. The progression-free survival (PFS) was the primary endpoint of this study; it was defined as the interval between the date of diagnosis and disease progression or death from any cause. The secondary endpoints were overall survival (OS), local relapse-free survival (LRFS), regional relapse-free survival (RRFS), locoregional relapse-free survival (LRRFS), and distant metastasis-free survival (DMFS), which were defined as the interval between diagnosis and death from any cause or the first event.

### Statistical analysis

Categorical variables were assessed using Fisher’s exact test and the Chi square test. Kaplan–Meier survival curves were used to analyze the time-to-event endpoints, and the log-rank test was used to compare the differences between groups. The hazard ratios (HRs) were calculated with the Cox proportional hazards model. Multivariable analyses were performed using the Cox proportional hazards model to test the independent statistical significance of treatment intervention. Potentially important prognostic factors considered in the modeling process included pretreatment plasma EBV DNA level, preoperative plasma EBV DNA level, and postoperative pathology. Analyses were performed using SPSS 22.0 (SPSS, Chicago, IL, USA). All statistical tests were two-sided, and *P* < 0.05 indicates statistical significance.

## Results

### Patient characteristics

Between January 2006 and December 2014, 292 NPC patients underwent neck dissection at the Department of Head and Neck Surgery at Sun Yat-sen University Cancer Center. Of these patients, 91 were suspected of having residual cervical lymphadenopathy. Clinical data of initial treatment were not available for 9 patients who had completed radical radiotherapy at other hospitals; these patients were therefore excluded. The remaining 82 patients met all the eligibility criteria and were selected for the analysis: 68 with tumor cells and 14 without tumor cells in dissected cervical lymph nodes as detected with postoperative pathological examination. Table 1 summarizes the characteristics of both groups. The median patient age was 43.5 (21–77) years in 68 patients with tumor cells and 46 (25–61) years in 14 patients without tumor cells in dissected cervical lymph nodes. The Chi square test revealed that female patients (*P* < 0.001) and patients with detectable EBV DNA before surgery (*P* = 0.045) were more likely to have tumor cells in dissected cervical lymph nodes.

**Table 1:** Characteristics of 82 NPC patients with residual cervical lymphadenopathy

Characteristic	Tumor cells in cervical lymph nodes [cases (%)]		*P* value
	Presence	Absence	
Total	68	14	
Sex			0.045^¶^
Male	52 (76.5)	7 (50.0)	
Female	16 (23.5)	7 (50.0)	
Pathological type			0.133^&^
WHO type II	2 (2.9)	2 (14.3)	
WHO type III	66 (97.1)	12 (85.7)	
T stage^a^			0.334^#^
T1	0 (0)	1 (7.1)	
T2	24 (35.3)	4 (28.6)	
T3	35 (51.5)	7 (50.0)	
T4	9 (13.2)	2 (14.3)	
N stage^a^			0.974^#^
N1	13 (19.1)	3 (21.4)	
N2	34 (50.0)	7 (50.0)	
N3	21 (30.9)	4 (28.6)	
Clinical stage^a^			0.179^¶^
II	7 (10.3)	4 (28.6)	
III	34 (50.0)	5 (35.7)	
IV	27 (39.7)	5 (35.7)	
EBV DNA before treatment (copies/mL)			0.252^¶^
≤ 4000	23 (33.8)	7 (50.0)	
> 4000	45 (66.2)	7 (50.0)	
EBV DNA before surgery (copy/mL) < 0.001^#^			
0	32 (47.1)	14 (100)	
> 0	36 (52.9)	0 (0.0)	
VCA-IgA			0.988^¶^
< 1:320	29 (42.6)	6 (42.9)	
≥ 1:320	39 (57.4)	8 (57.1)	
EA-IgA			0.196^¶^
< 1:20	27 (39.7)	3 (21.4)	
≥ 1:20	41 (60.3)	11 (78.6)	
Size of lymph node 0.747^¶^			
< 3 cm	42 (61.8)	8 (57.1)	
≥ 3 cm	26 (38.2)	6 (42.9)	
Lymph node invasion 1.000^#^			
Yes	4 (5.9)	0 (0.0)	
No	64 (94.1)	14 (100)	
Lymph node necrosis 0.865^¶^			
Yes	16 (23.5)	3 (21.4)	
No	52 (76.5)	11 (78.6)	
Treatment for cervical lymphadenopathy			0.285^#^
Surgery alone	54 (79.4)	14 (100)	
Surgery + chemotherapy	9 (13.2)	0 (0.0)	
Surgery + radiotherapy	5 (7.5)	0 (0.0)	
Treatment for NPC			0.957^#^
Radiotherapy alone	4 (5.9)	1 (7.15)	
CCRT	22 (32.4)	5 (35.7)	
NAC + CCRT	38 (55.9)	7 (50.0)	
CCRT + AC	4 (5.9)	1 (7.15)	
Irradiation technique			1.000^&^
2D-RT	7 (10.3)	1 (7.1)	
IMRT	61 (89.7)	13 (92.9)	

*NPC* nasopharyngeal carcinoma, *WHO* World Health Organization, *EBV* Epstein-Barr virus, *VCA* viral capsid antigen, *IgA* immunoglobulin A, *EA* early antigen, *2D*-*RT* two-dimensional radiotherapy, *IMRT* intensity-modulated radiotherapy, *CCRT* concurrent chemoradiotherapy, *NAC* neoadjuvant chemotherapy, *AC* adjuvant chemotherapy

*P* values were calculated with ^¶^the Chi square test, ^&^Continuity Correlation, and ^#^the Fisher’s exact test, respectively

^a^The 7th edition of American Joint Committee on Cancer/Union for International Cancer Control (AJCC/UICC) staging system was used

### Clinical analyses of plasma EBV DNA levels in NPC patients

**Fig. 1:**
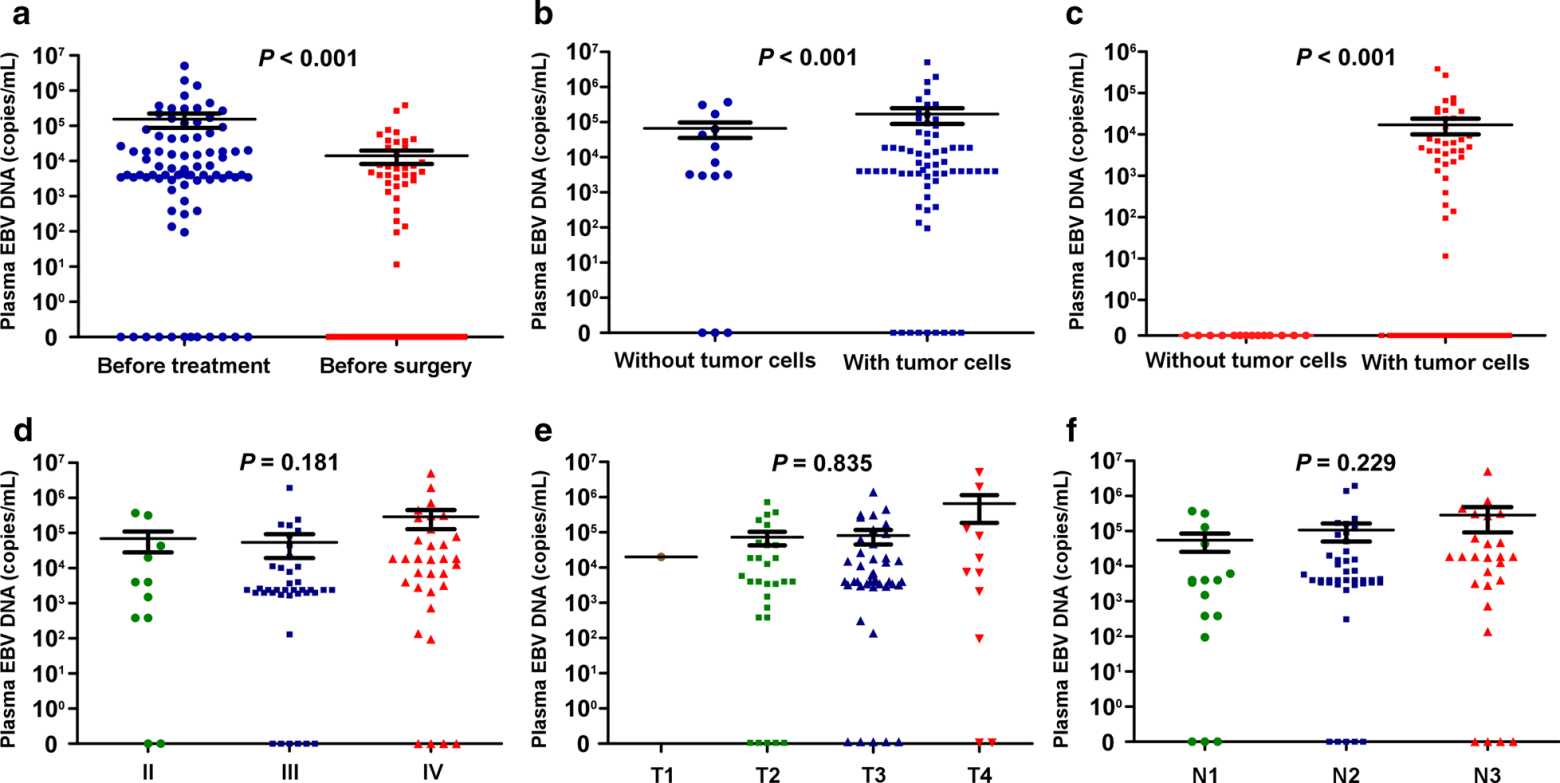
Plasma Epstein-Barr virus (EBV) DNA levels in patients with nasopharyngeal carcinoma (NPC). **a** Plasma EBV DNA levels before the initiation of treatment and before surgery for all 82 patients; **b** plasma EBV DNA levels before the initiation of treatment for the patients with and without tumor cells detected in dissected cervical lymph nodes; **c** plasma EBV DNA levels before neck dissection for the patients with and without tumor cells detected in dissected cervical lymph nodes; **d** plasma EBV DNA levels before the initiation of treatment for all 82 patients according to clinical stage; **e** plasma EBV DNA levels before the initiation of treatment for all 82 patients according to T stage; **f** plasma EBV DNA levels before the initiation of treatment for all 82 patients according to N stage

Using real-time qPCR, we detected EBV DNA levels in plasma samples from the patients before the initiation of treatment and before neck dissection. The median plasma EBV DNA level before the initiation of treatment was 4160 copies/mL (interquartile range, 2652–44,400 copies/mL) for all 82 NPC patients (Fig. 1a) and 117 copies/mL (interquartile range, 0–6260 copies/mL) for the 68 patients with tumor cells in dissected cervical lymph nodes (Fig. 1b). In addition, all 14 patients without tumor cells in dissected cervical lymph nodes had undetectable plasma EBV DNA levels before neck dissection (Fig. 1c). The median plasma EBV DNA level was not associated with clinical stage (Fig. 1d), T stage (Fig. 1e), and N stage (Fig. 1f).

### The sensitivity and specificity of preoperative EBV DNA detection and FNAC of cervical lymph nodes in identifying residual cervical lymphadenopathy

The preoperative plasma EBV DNA level was detectable in 36 patients and undetectable in 46 patients. Due to poor patient compliance, FNAC of cervical lymph nodes was only performed in 21 patients, all of whom underwent intraoperative frozen section examination. FNAC results showed that 16 (76.1%) were positive and 5 (23.9%) were negative for malignant cells in cervical lymph nodes. Of the 5 patients with negative FNAC results, 3 had positive postoperative pathological findings for residual cervical lymphadenopathy (1 had detectable preoperative plasma EBV DNA levels and 2 had undetectable preoperative plasma EBV DNA levels). Thus, the sensitivity and specificity of FNAC in identifying residual cervical lymphadenopathy were 84.2% (16/19) and 100% (2/2), whereas those of preoperative EBV DNA detection were 52.9% (36/68) and 100% (14/14). The sensitivity of FNAC combined with preoperative EBV DNA detection increased to 89.5% (17/19) (Table 2).

**Table 2:** The sensitivity, specificity, PPV, and NPV of preoperative examinations in identifying residual cervical lymphadenopathy in patients with NPC

Examination	Sensitivity (%)	Specificity (%)	PPV (%)	NPV (%)
FNAC	84.2	100.0	100.0	40.0
EBV DNA detection	52.9	100.0	100.0	30.4
FNAC + EBV DNA detection	89.5	100.0	100.0	40.0

*PPV* positive predictive value, *NPV* negative predictive value, *NPC* nasopharyngeal carcinoma, *FNAC* fine needle aspiration cytology, *EBV* Epstein-Barr virus

### Survival analysis based on postoperative pathological results

**Fig. 2:**
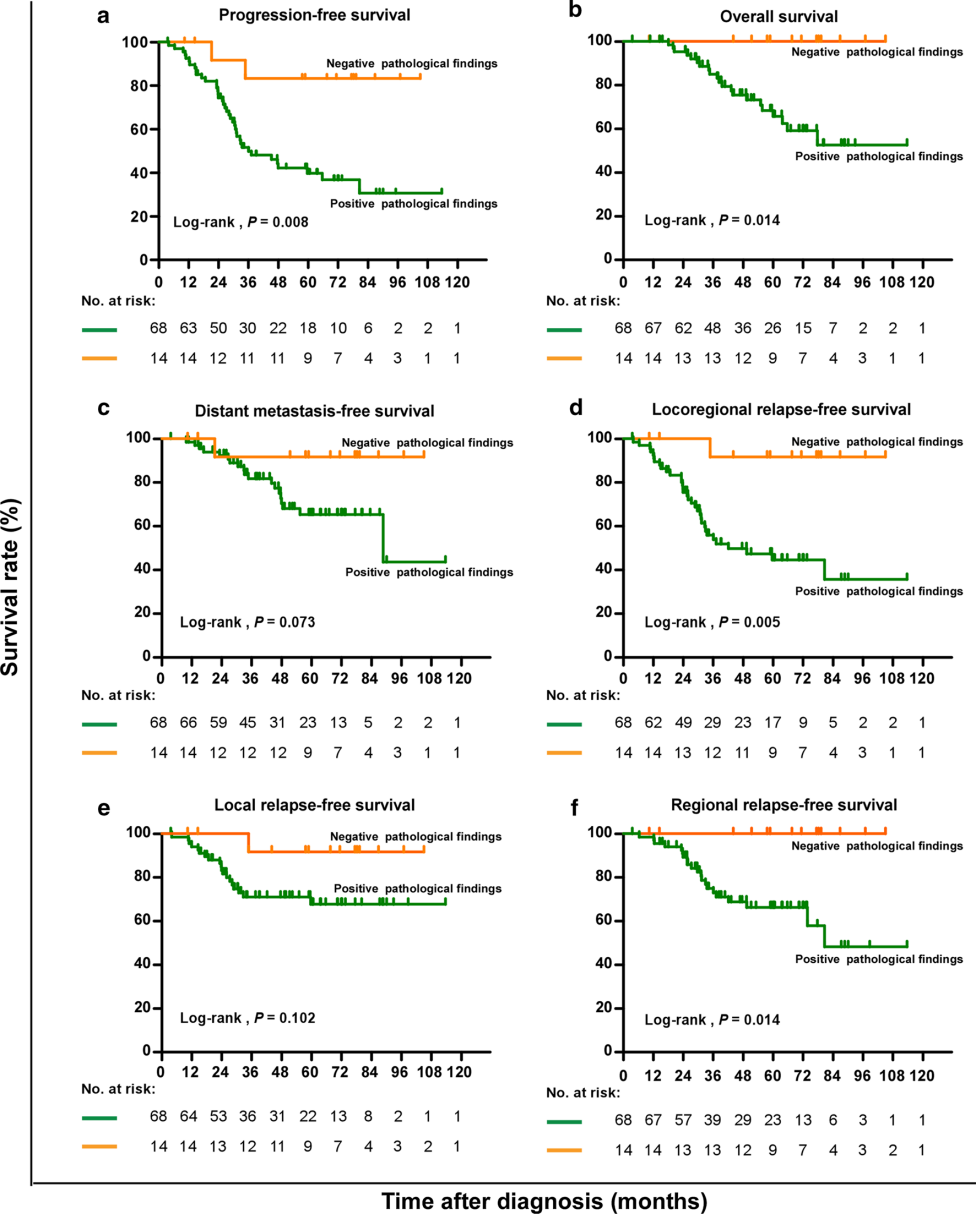
Kaplan-Meier estimates of the survival of NPC patients. Of the 82 patients, 68 had positive postoperative pathological results, and 14 had negative postoperative pathological results. **a** Progression-free survival; **b** overall survival; **c** distant metastasis-free survival; **d** locoregional relapse-free survival; **e** Local relapse-free survival; and **f** Regional relapse-free survival

The median follow-up period for the entire patient cohort was 52.6 (interquartile range, 3.6–116.5) months. During the follow-up period, 41 of the 82 patients experienced disease progression: 39 (95.1%) with positive postoperative pathological results and 2 (4.9%) with negative postoperative pathological results. Twenty (24.4%) patients died, and all of them had positive postoperative pathological findings. However, one of the patients died in a car accident, and 19 (95.0%) of the deaths were reported as being disease-related. Kaplan–Meier survival analysis revealed that a positive postoperative pathological finding was significantly associated with shorter PFS (*P *= 0.008), OS (*P* = 0.014), LRRFS (*P* = 0.005), and RRFS (*P* = 0.014), but not with DMFS (*P* = 0.073) and LRFS (*P* = 0.102) (Table 3, Fig. 2).

**Table 3:** Survival of the 82 NPC patients with residual cervical lymphadenopathy

Endpoint	Tumor cells in cervical lymph nodes		*P* value	Preoperative plasma EBV DNA		*P* value
	Presence	Absence		Presence	Absence	
Total (cases)	68	14		36	46	
PFS						
Failures [cases (%)]	39 (57.4)	2 (14.3)	0.008	22 (61.1)	19 (41.3)	0.031
3-year rate [% (95% CI)]	49.9 (37.6–62.2)	83.3 (62.1–100)		43.7 (26.1–61.3)	61.1 (46.6–75.6)	
OS						
Deaths [cases (%)]	21 (30.9)	0 (0.0)	0.014	13 (36.1)	8 (17.4)	0.020
3-year rate [% (95% CI)]	83.2 (73.6–92.8)	100		79.2 (64.3–94.1)	90.7 (82.1–99.3)	
DMFS						
Failures [cases (%)]	19 (27.9)	1 (7.1)	0.073	10 (27.8)	10 (21.7)	0.230
3-year rate [% (95% CI)]	79.6 (69.2–90.0)	91.7 (76.0–100)		80.2 (65.9–94.5)	85.9 (75.3–96.5)	
LRRFS						
Failures [cases (%)]	33 (48.5)	1 (7.1)	0.005	20 (55.6)	14 (30.4)	0.009
3-year rate [% (95% CI)]	53.9 (41.2–66.6)	91.7 (76.0–100)		46.4 (28.4–64.4)	68.2 (53.7–82.7)	
LRFS						
Failures [cases (%)]	19 (27.9)	1 (7.1)	0.102	13 (36.1)	7 (15.2)	0.017
3-year rate [% (95% CI)]	71.0 (59.6–82.4)	91.7 (76.0–100)		61.1 (44.4–77.8)	84.9 (73.7–96.1)	
RRFS						
Failures [cases (%)]	21 (30.9)	0 (0.0)	0.014	13 (36.1)	8 (17.4)	0.027
3-year rate [% (95% CI)]	73.0 (61.6–84.4)	100		69.8 (52.9–86.7)	80.7 (68.5–92.9)	

*NPC* nasopharyngeal carcinoma, *PFS* progression-free survival, *OS* overall survival, *DMFS* distant metastasis-free survival, *LRRFS* locoregional relapse-free survival, *LRFS* local relapse-free survival, *RRFS* regional relapse-free survival, *CI* confidence interval

*P* values were calculated with the unadjusted log-rank test

### Survival analysis based on preoperative plasma EBV DNA level

**Fig. 3:**
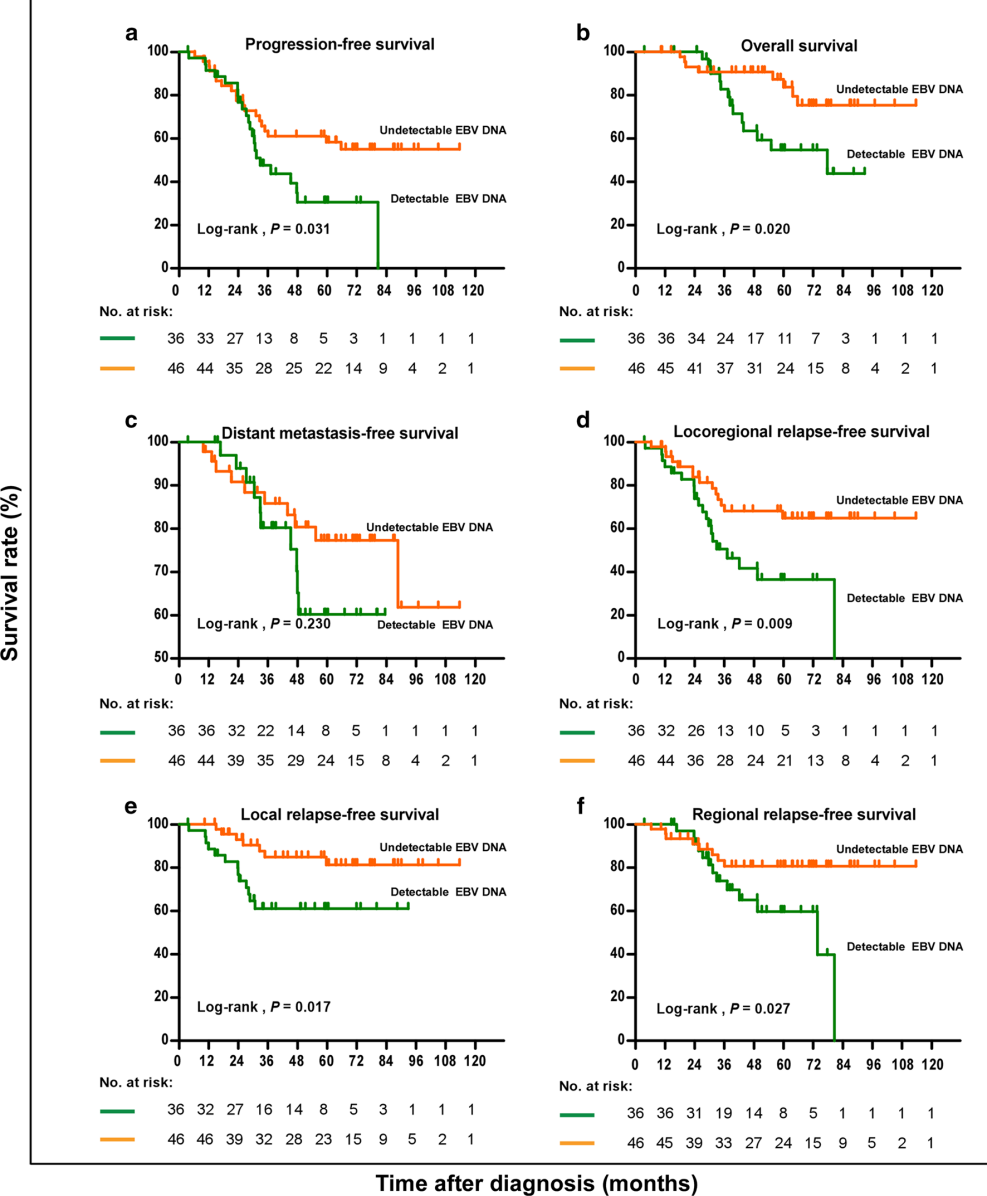
Kaplan-Meier estimates of the survival of NPC patients with residual cervical lymphadenopathy. Of the 82 patients, 36 had detectable preoperative plasma EBV DNA, and 46 had undetectable preoperative plasma EBV DNA. **a** Progression-free survival; **b** overall survival; **c** distant metastasis-free survival; **d** locoregional relapse-free survival; **e** local relapse-free survival; and **f** regional relapse-free survival

Given that plasma EBV DNA is the most effective predictive biomarker in guiding the treatment and predicting the prognosis of NPC, we analyzed the efficacy of plasma EBV DNA detection in identifying residual cervical lymphadenopathy in NPC patients. Compared with the 46 patients without detectable preoperative plasma EBV DNA, the 36 patients with detectable preoperative plasma EBV DNA demonstrated significantly lower 3-year PFS, OS, LRRFS, LRFS, and RRFS rates (all *P* < 0.05), but did not demonstrate significantly lower 3-year DMFS rate (Table 3). The same associations between preoperative plasma EBV DNA and survival were observed in the patients with residual cervical lymphadenopathy (Fig. 3).

### Prognostic factors for NPC patients with residual cervical lymphadenopathy

The multivariate analysis showed that postoperative pathological results remained an independent predictor of short PFS (HR = 5.209, 95% CI 1.185–22.900; *P* = 0.029), DMFS (HR = 9.265, 95% CI 1.035–82.935; *P* = 0.047), and LRFFS (HR = 10.175, 95% CI 1.273–81.320; *P* = 0.029), and the presence of preoperative EBV DNA remained an independent predictor of short OS (HR = 5.535, 95% CI 1.677–18.268; *P* = 0.005) and RRFS (HR = 2.804, 95% CI 1.018–7.727; *P* = 0.046) for the 82 patients (Table 4). To further assess the prognostic value of preoperative EBV DNA, the patients were stratified into subgroups based on postoperative pathological results. Multivariate analysis revealed that the presence of preoperative plasma EBV DNA remained an independent predictor of short OS in patients with positive postoperative pathological results (HR = 3.501, 95% CI 1.075–11.398; *P* = 0.037) (Table 5). These results indicate that preoperative plasma EBV DNA represents a valuable independent prognostic factor for NPC patients with residual cervical lymphadenopathy.

**Table 4:** Multivariable analysis of prognostic factors of all 82 NPC patients with residual cervical lymphadenopathy

Variable	PFS		OS		DMFS		LRRFS		LRFS		RRFS	
	HR (95% CI)	*P* value	HR (95% CI)	*P* value	HR (95% CI)	*P* value	HR (95% CI)	*P* value	HR (95% CI)	*P* value	HR (95% CI)	*P* value
Age	0.827 (0.408–1.675)	0.598	1.604 (0.625–4.113)	0.326	0.440 (0.148–1.308)	0.140	0.728 (0.331–1.600)	0.429	0.619 (0.224–1.715)	0.357	1.328 (0.507–3.479)	0.564
Sex	0.699 (0.336–1.454)	0.338	0.503 (0.164–1.542)	0.230	0.467 (0.148–1.472)	0.194	0.533 (0.237–1.197)	0.127	0.531 (0.189–1.492)	0.230	0.585 (0.211–1.619)	0.302
T stage	2.739 (1.173–6.397)	0.020	4.114 (1.140–14.847)	0.031	10.722 (2.408–47.740)	0.002	1.536 (0.611–3.861)	0.361	1.433 (0.437–4.702)	0.553	2.606 (0.803–8.465)	0.111
N stage	2.735 (0.761–9.837)	0.123	2.530 (0.577–11.090)	0.218	9.408 (1.465–60.435)	0.018	1.475 (0.367–5.927)	0.583	2.379 (0.359–15.749)	0.369	2.085 (0.416–10.446)	0.372
Clinical stage	0.516 (0.214–1.246)	0.142	1.309 (0.421–4.071)	0.642	0.384 (0.095–1.549)	0.179	0.637 (0.266–1.530)	0.313	0.408 (0.120–1.392)	0.152	0.740 (0.244–2.242)	0.594
EBV DNA before first treatment	1.360 (0.658–2.811)	0.407	0.777 (0.276–2.184)	0.632	1.639 (0.548–4.901)	0.377	1.912 (0.843–4..338)	0.121	1.000 (0.357–2.798)	0.999	3.576 (1.010–12.658)	0.048
Preoperative EBV DNA	1.546 (0.758–3.151)	0.231	5.535 (1.677–18.268)	0.005	1.898 (0.655–5.499)	0.238	1.571 (0.733–3.365)	0.245	2.622 (0.944–7.286)	0.064	2.804 (1.018–7.727)	0.046
Postoperative pathological result	5.209 (1.185–22.900)	0.029	–	–	9.265 (1.035–82.935)	0.047	10.175 (1.273–81.320)	0.029	4.158 (0.457–37.820)	0.206	–	–
Treatment for NPC	1.282 (0.780–2.108)	0.327	1.702 (0.711–4.070)	0.232	2.619 (0.929–7.383)	0.069	1.141 (0.672–1.938)	0.626	0.865 (0.476–1.573)	0.635	2.398 (0.971–5.922)	0.058

*NPC* nasopharyngeal carcinoma, *PFS* progression-free survival, *OS* overall survival, *DMFS* distant metastasis-free survival, *LRRFS* locoregional relapse-free survival, *LRFS* local relapse-free survival, *RRFS* regional relapse-free survival, *HR* hazard ratio, *CI* confidence interval

All variables were transformed into categorical variables. HRs were calculated for age (≥ 45 vs. < 45 years), sex (male vs. female), T stage (3–4 vs. 1–2), N stage (3 vs. 1–2), clinical stage (IV vs. III vs. II), EBV DNA before first treatment (> 4000 vs. ≤ 4000 copies/mL), preoperative EBV DNA (> 0 vs. 0 copy/mL), postoperative pathology (positive vs. negative), and treatment for NPC (RT vs. CCRT vs. NAC + CCRT vs. CCRT + AC)

**Table 5:** Multivariable analysis of prognostic factors of 68 NPC patients with positive postoperative pathological results

Variable	PFS		OS		DMFS		LRRFS		LRFS		RRFS	
	HR (95% CI)	*P* value	HR (95% CI)	*P* value	HR (95% CI)	*P* value	HR (95% CI)	*P* value	HR (95% CI)	*P* value	HR (95% CI)	*P* value
Age	0.948 (0.458–1.962)	0.885	1.365 (0.501–3.719)	0.543	0.505 (0.164–1.555)	0.234	0.750 (0.336–1.676)	0.483	0.662 (0.231–1.895)	0.442	1.097 (0.412–2.917)	0.853
Sex	0.866 (0.401–1.871)	0.715	0.506 (0.160–1.600)	0.246	0.652 (0.192–2.208)	0.492	0.568 (0.248–1.302)	0.182	0.595 (0.202–1.752)	0.346	0.489 (0.176–1.360)	0.171
T stage	2.733 (1.139–6.557)	0.024	4.523 (1.240–16.501)	0.022	11.418 (2.406–54.182)	0.002	1.616 (0.633–4.126)	0.315	1.473 (0.441–4.923)	0.529	2.985 (0.900–9.894)	0.074
N stage	2.070 (0.555–7.726)	0.279	2.852 (0.564–14.409)	0.205	8.385 (1.213–57.978)	0.031	1.380 (0.339–5.617)	0.653	2.054 (0.303–13.919)	0.461	2.979 (0.528–16.798)	0.216
Clinical stage	0.593 (0.240–1.463)	0.256	1.183 (0.369–3.790)	0.777	0.361 (0.086–1.515)	0.164	0.696 (0.285–1.697)	0.425	0.467 (0.132–1.651)	0.237	0.621 (0.192–2.008)	0.426
EBV DNA before first treatment	1.166 (0.553–2.459)	0.687	0.812 (0.287–2.295)	0.695	1.296 (0.287–0.423)	0.650	1.762 (0.770–4.033)	0.180	0.868 (0.300–2.510)	0.793	3.631 (1.013–13.019)	0.048
Preoperative EBV DNA	1.589 (0.768–3.290)	0.212	3.501 (1.075–11.398)	0.037	2.051 (0.688–6.119)	0.197	1.575 (0.732–3.389)	0.246	2.588 (0.926–7.234)	0.070	1.827 (0.684–4.882)	0.229
Treatment for NPC	1.341 (0.800–2.247)	0.266	1.621 (0.684–3.845)	0.273	3.367 (1.081–10.489)	0.036	1.120 (0.657–1.910)	0.677	0.840 (0.461–1.531)	0.569	2.184 (0.874–5.459)	0.095

*NPC* nasopharyngeal carcinoma, *PFS* progression-free survival, *OS* overall survival, *DMFS* distant metastasis-free survival, *LRRFS* locoregional relapse-free survival, *LRFS* local relapse-free survival, *RRFS* regional relapse-free survival, *HR* hazard ratio, *CI* confidence interval

All variables were transformed into categorical variables. HRs were calculated for age (≥ 45 vs. < 45 years), sex (male vs. female), T stage (3–4 vs. 1–2), N stage (3 vs. 1–2), clinical stage (IV vs. III vs. II), EBV DNA before first treatment (> 4000 vs. ≤ 4000 copies/mL), preoperative EBV DNA (> 0 vs. 0 copy/mL), and treatment for NPC (RT vs. CCRT vs. NAC + CCRT vs. CCRT + AC)

## Discussion

In the present study, 68 (82.9%) of the 82 NPC patients suspected of having residual cervical lymphadenopathy exhibited positive postoperative pathological results. Additionally, a positive postoperative pathological result was significantly associated with low 3-year PFS, OS, LRRFS, and RRFS rates and was confirmed as an independent prognostic factor for PFS. Furthermore, our study showed that using FNAC combined with preoperative EBV DNA detection improved the sensitivity in diagnosing NPC with residual cervical lymphadenopathy.

Persistent nodal disease in NPC patients after definitive radiotherapy presents a diagnostic and treatment challenge in clinical practice. In these patients, tumor cells are often not observed in the neck dissection specimens. Previous studies have demonstrated that 58.3%–88.2% of NPC patients presenting symptoms of persistent/recurrent neck mass had cervical malignancies [26, 27]. It is thus imperative to define the nature of the presumed persistent neck mass before surgery to avoid unnecessary treatment.

Currently, there is no well-accepted method for preoperative determination of the presence of malignancy in nodal diseases, although it was reported that FNAC was helpful in differentiating malignant lymphadenopathy from benign inflammatory nodes [28, 29]. In clinical practice, the confirmation of cervical malignancy may still require some surgical intervention. One study has shown that the sensitivity and specificity of FNAC in identifying malignant lymphadenopathy were 25% and 100% [26]. In the present study, the sensitivity and specificity of FNAC in identifying cervical lymphadenopathy were 84.2% and 100%. Furthermore, we demonstrated that the sensitivity and specificity of preoperative EBV DNA detection in identifying cervical lymphadenopathy were 52.9% and 100%, and those of FNAC combined with preoperative EBV-DNA detection were 89.5% and 100%. The diagnostic efficacy of FNAC for residual cervical lymphadenopathy in NPC patients after radiotherapy is significantly reduced. For early and correct diagnosis, FNAC combined with preoperative EBV DNA detection should be adopted. The present findings indicate that if EBV DNA is detectable before surgery, the patients are more likely to have a positive postoperative pathological result.

NPC patients with residual cervical lymphadenopathy presented with a higher degree of heterogeneity than did patients without residual cervical lymphadenopathy. The prognostic significance of TNM classification, which only reflects anatomical information, is not optimal for NPC patients with residual cervical lymphadenopathy. Multiple studies have demonstrated that EBV DNA serves as a reliable biomarker in the detection, monitoring, and prognostic prediction for NPC [21, 22, 25, 30–37]. Moreover, the presence of EBV DNA after radiotherapy is the most important independent prognostic marker in predicting survival and outcome; the prognosis of patients with detectable EBV DNA after radiotherapy was significantly worse than that of those with undetectable EBV DNA [38]. Using a cutoff > or = 0 copy/mL, we report that the presence of preoperative EBV DNA was associated with low 3-year OS, PFS, LRRFS, LRFS, and RRFS rates. Furthermore, detectable preoperative EBV DNA was confirmed as an independent prognostic factor for OS and PFS in both the entire cohort and the patients with positive postoperative pathological results. Although the DMFS rates were not significantly different between patients with detectable and undetectable preoperative plasma EBV DNA, the survival curves showed that the patients with detectable preoperative plasma EBV DNA had a higher risk of distant metastasis. In the present study, all 36 NPC patients with residual cervical lymphadenopathy who had detectable preoperative EBV DNA underwent neck dissection, but still had poor prognosis. These findings suggest that neck dissection alone is not sufficient for these high-risk patients and that these patients may require a more aggressive treatment strategy.

For high-risk NPC patients, a more intensive treatment regimen such as AC can provide an additional survival benefit over neck dissection alone. In addition, AC can kill tumor cells that might have remained following macroscopic tumor removal and eliminate micrometastasis. Even though previous meta-analyses revealed that there was no benefit of using AC for NPC patients [7, 39] and all previous trials on AC had failed, Twu et al. [40] demonstrated that AC reduced distant failure and prolonged OS in NPC patients with persistently detectable EBV DNA after curative radiotherapy. In fact, the efficacy of AC is being addressed in ongoing trials which target patients with residual post-therapy EBV DNA. For these reasons, a prospective randomized trial comparing neck dissection combined with AC to neck dissection alone should be initiated to assess whether AC can effectively treat NPC patients with residual cervical lymphadenopathy who have detectable preoperative EBV DNA levels. The current NCCN guidelines recommend the PF regimen (cisplatin + 5-fluorouracil) as the standard AC regimen for advanced NPC according to the results of an intergroup study [3]. However, Zhang et al. [41] have shown that the GP regimen (gemcitabine + cisplatin) prolonged PFS in patients with recurrent or metastatic NPC, which established the GP regimen as the standard first-line treatment for this population. Furthermore, prospective randomized trials are strongly recommended to investigate the most effective AC regimen (PF vs. GP) for high-risk NPC patients.

The present study has several limitations. First, there is inevitable selection bias caused by its retrospective nature. Prospective studies are required to validate our results. Second, the sample size is small due to the rarity of these cases. Third, this was a single-center analysis from a high-prevalence district. A multi-center study is needed to fully evaluate the diagnostic and prognostic values of preoperative plasma EBV DNA detection in NPC patients with residual cervical lymphadenopathy.

## Conclusions

Using FNAC combined with preoperative EBV DNA detection could improve the sensitivity in identifying residual cervical lymphadenopathy in NPC patients. Compared with patients with undetectable EBV DNA, patients with detectable preoperative plasma EBV DNA may have worse prognosis. These patients require a more aggressive treatment strategy, and future trials should consider preoperative EBV DNA levels as a stratification factor and investigate the optimal regimen for the target population.

## Abbreviations

NPC: nasopharyngeal carcinoma
PFS: progression-free survival
OS: overall survival
DMFS: distant metastasis-free survival
LRRFS: locoregional relapse-free survival
LRFS: local relapse-free survival
RRFS: regional relapse-free survival
HR: hazard ratio
CI: confidence interval
CCRT: concurrent chemoradiotherapy
2D-RT: two-dimensional radiotherapy
IMRT: intensity-modulated radiotherapy
NCCN: National Comprehensive Cancer Network
MRI: magnetic resonance imaging
EBV: Epstein-Barr virus
WHO: World Health Organization
NAC: neoadjuvant chemotherapy
AC: adjuvant chemotherapy
FNAC: fine needle aspiration cytology
VCA: viral capsid antigen
IgA: immunoglobulin A
EA: early antigen
